# Supplementary figures and images for: Adult sex change leads to extensive forebrain reorganization in clownfish
Source: Biol Sex Differ. 2024 Jul 23;15:58. doi: 10.1186/s13293-024-00632-0 (PMC11267845; doi:10.1186/s13293-024-00632-0)

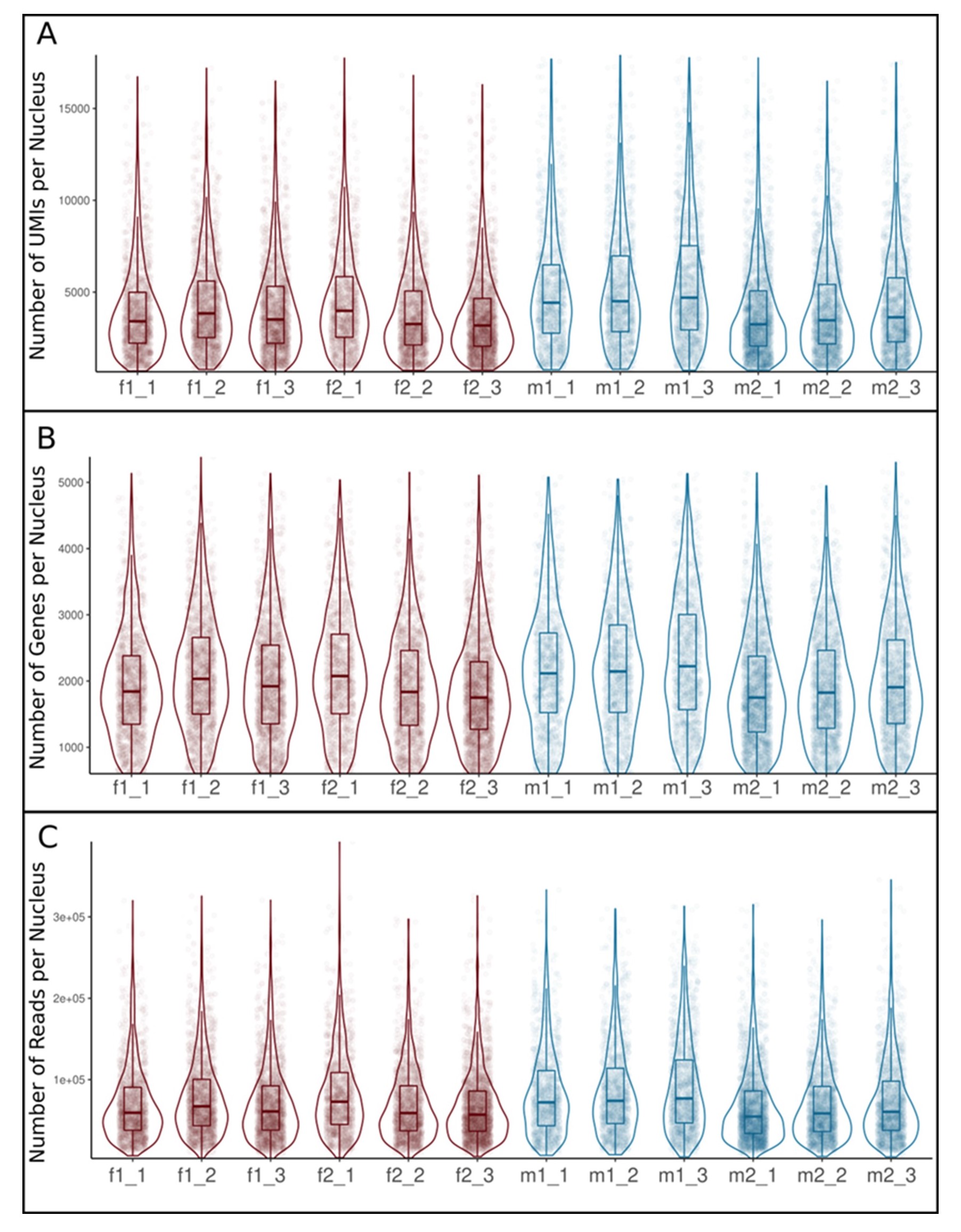

Supplement: Supplementary file 1 — Supplementary Material 1 [file 13293_2024_632_MOESM1_ESM.jpg]

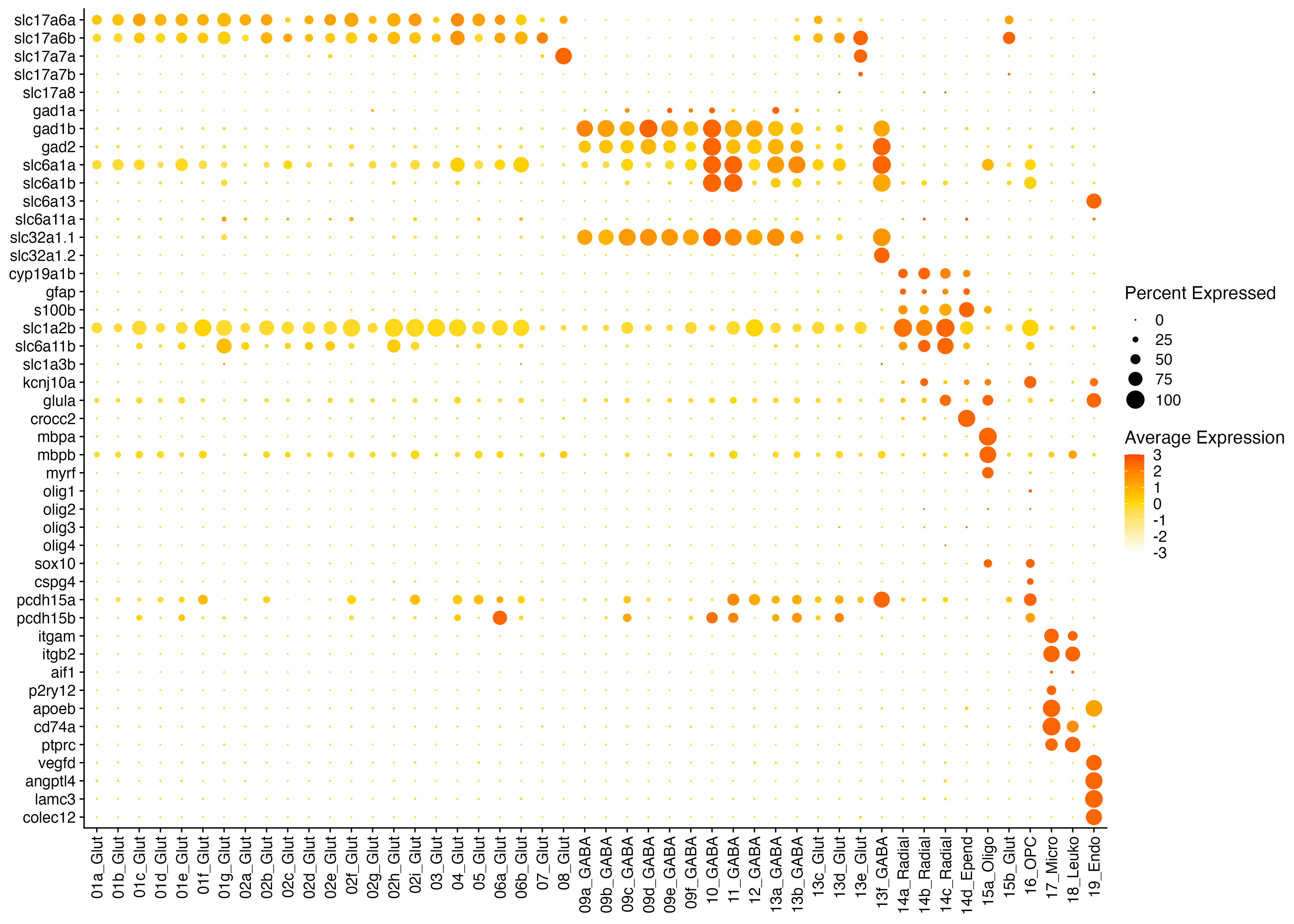

Supplement: Supplementary file 2 — Supplementary Material 2 [file 13293_2024_632_MOESM2_ESM.jpg]

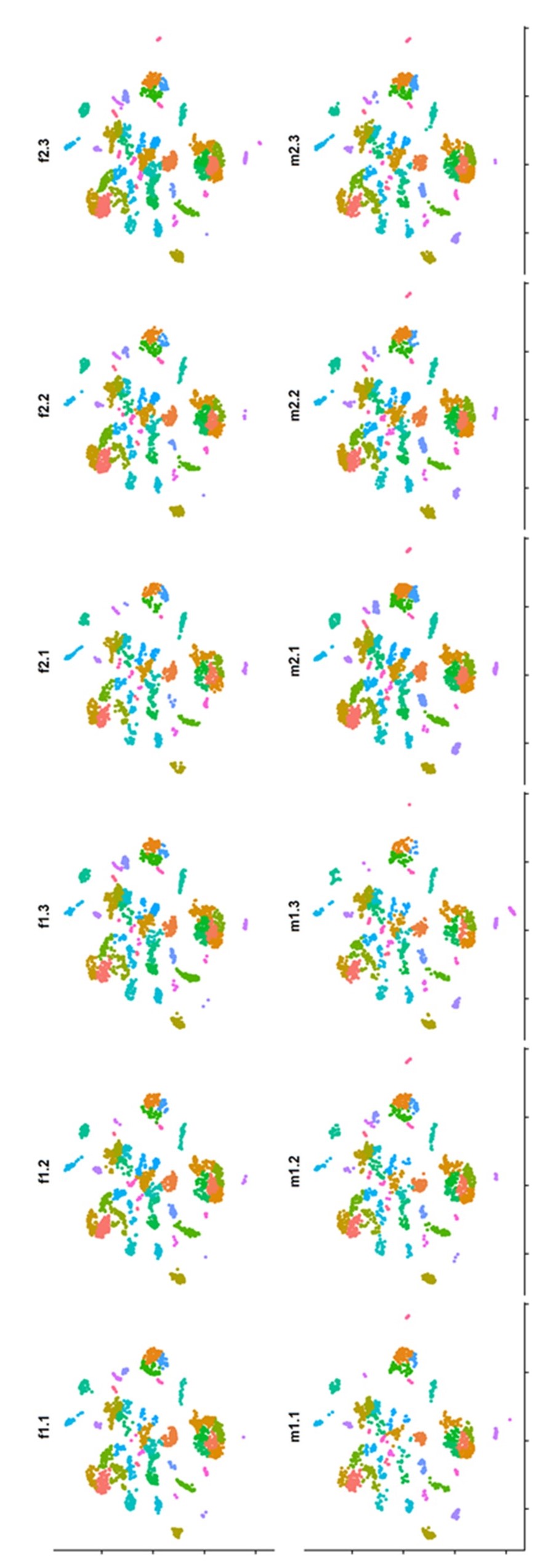

Supplement: Supplementary file 3 — Supplementary Material 3 [file 13293_2024_632_MOESM3_ESM.jpg]

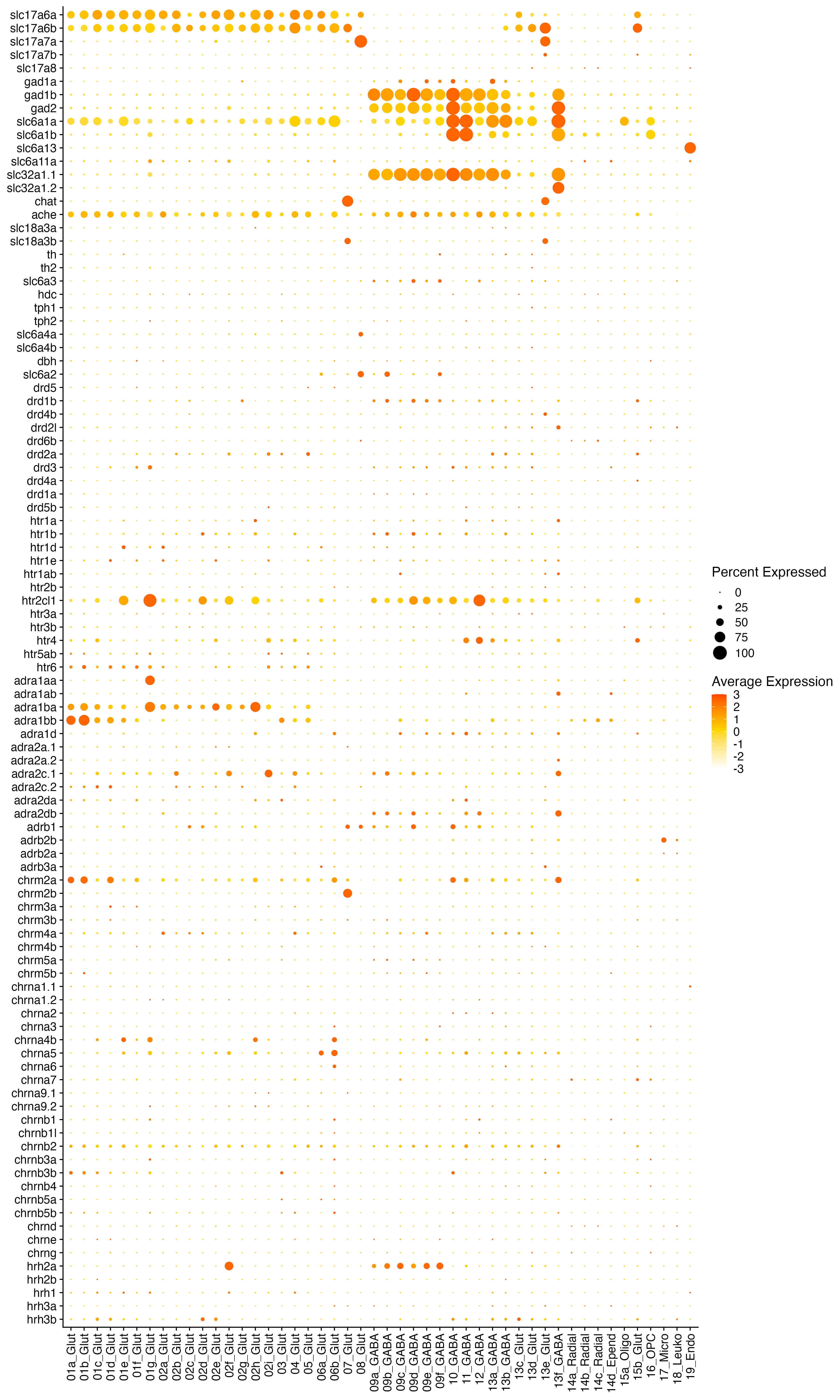

Supplement: Supplementary file 4 — Supplementary Material 4 [file 13293_2024_632_MOESM4_ESM.jpg]

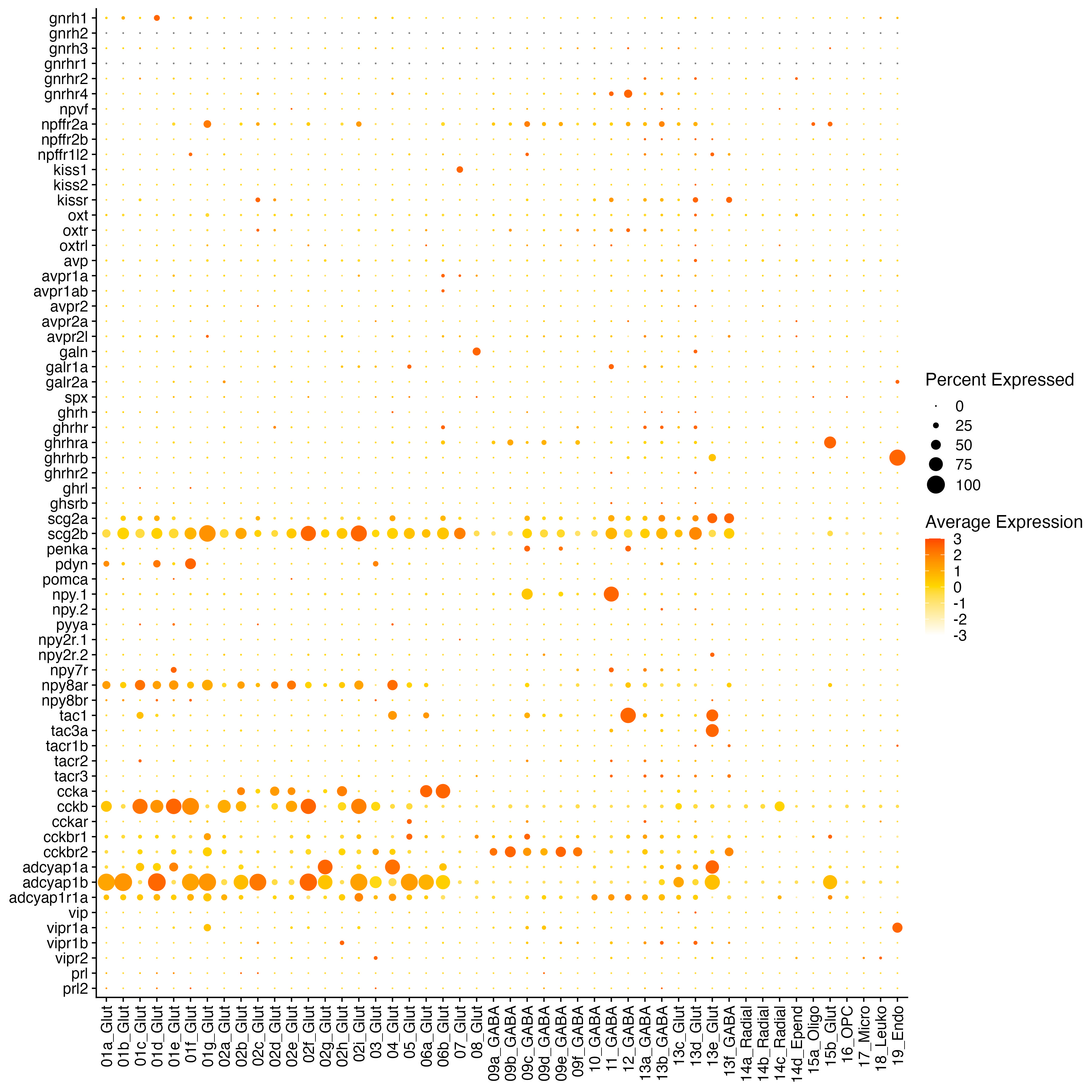

Supplement: Supplementary file 5 — Supplementary Material 5 [file 13293_2024_632_MOESM5_ESM.jpg]

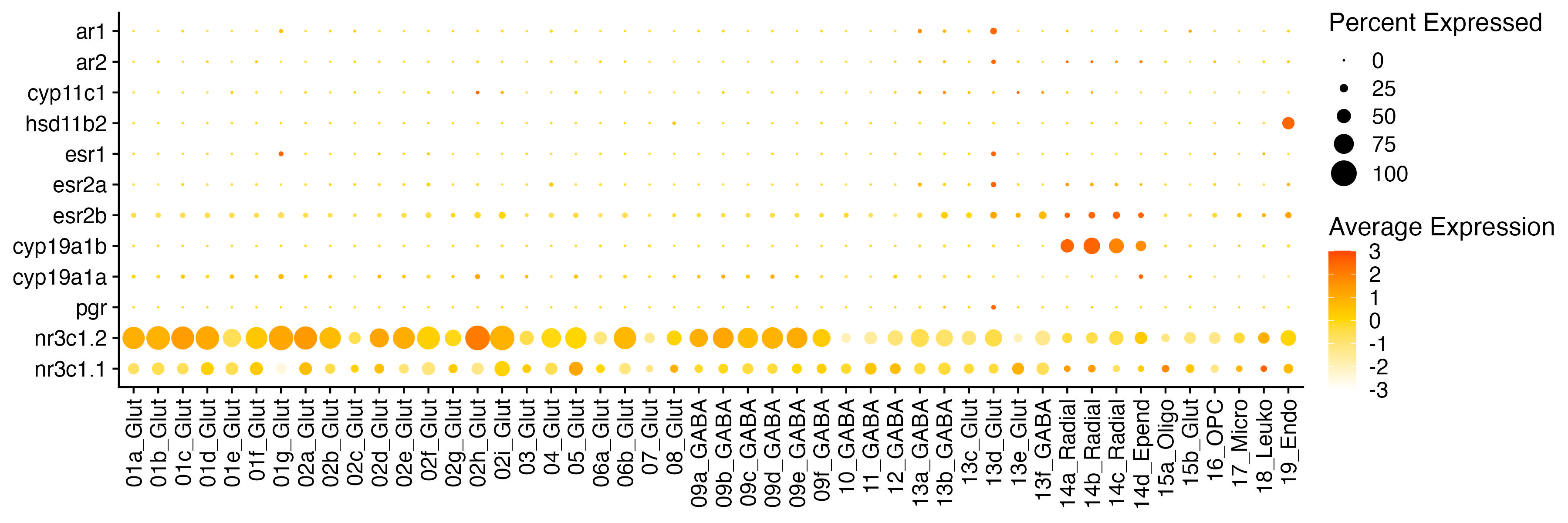

Supplement: Supplementary file 6 — Supplementary Material 6 [file 13293_2024_632_MOESM6_ESM.jpg]

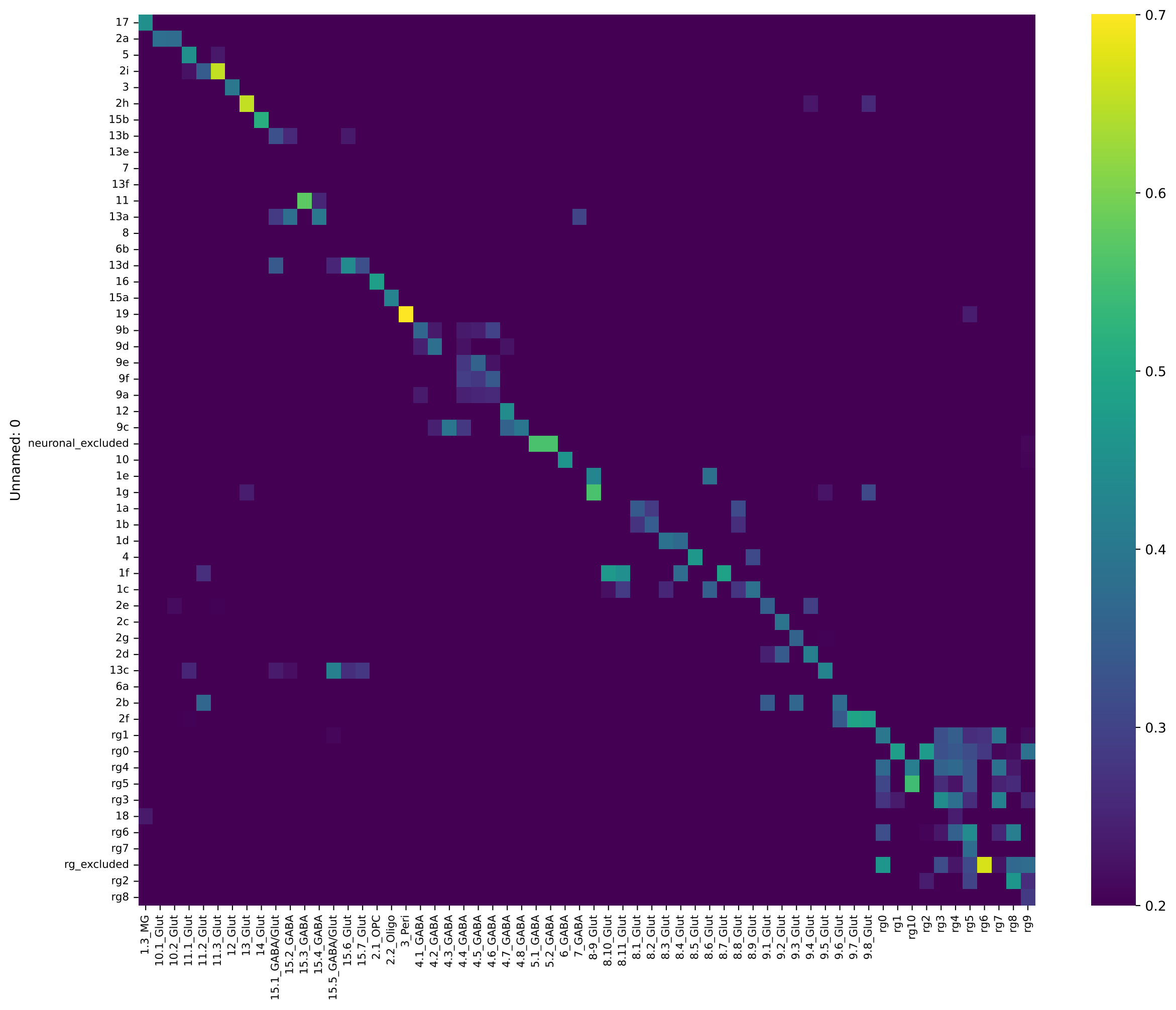

Supplement: Supplementary file 7 — Supplementary Material 7 [file 13293_2024_632_MOESM7_ESM.pdf]

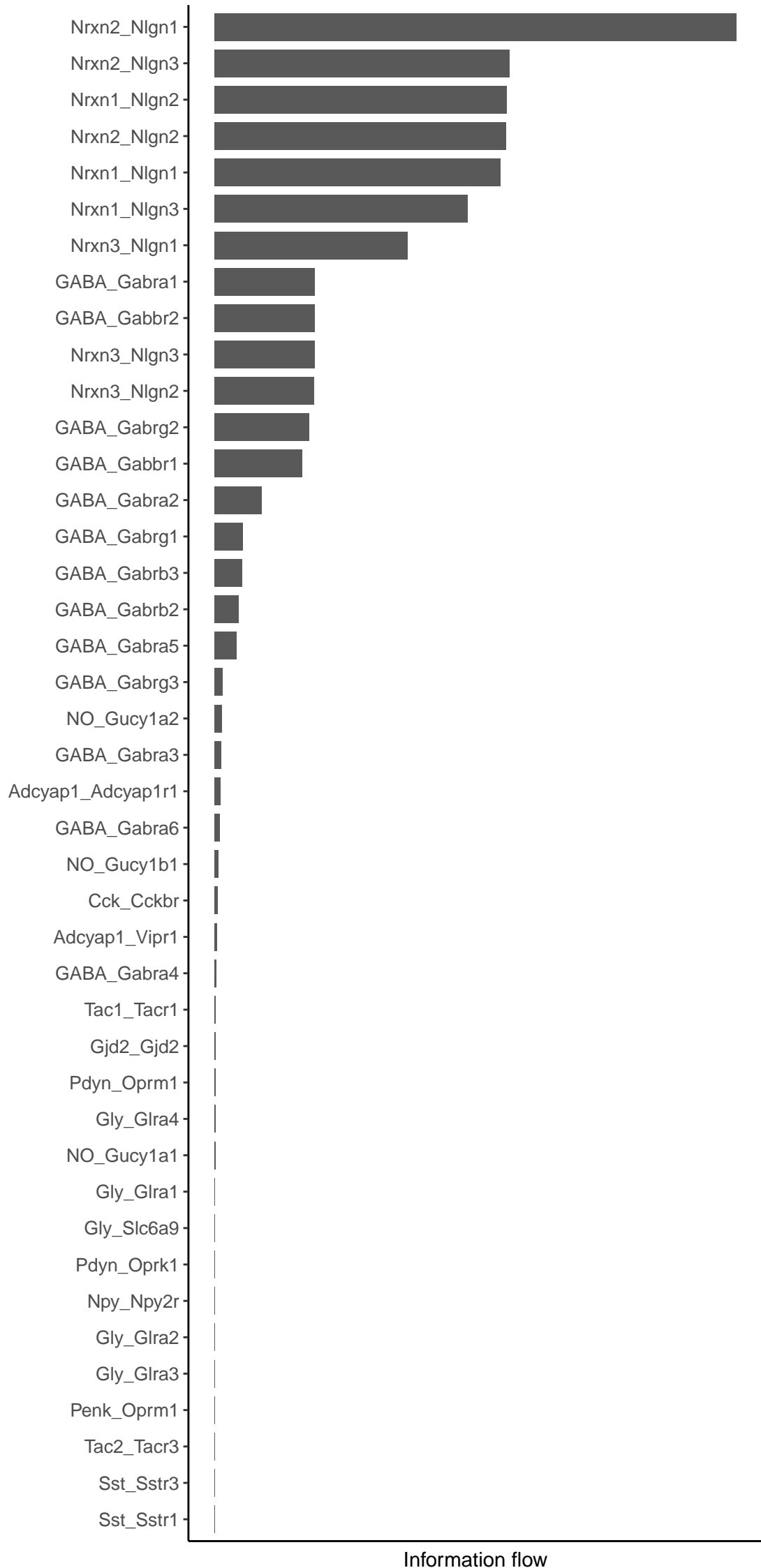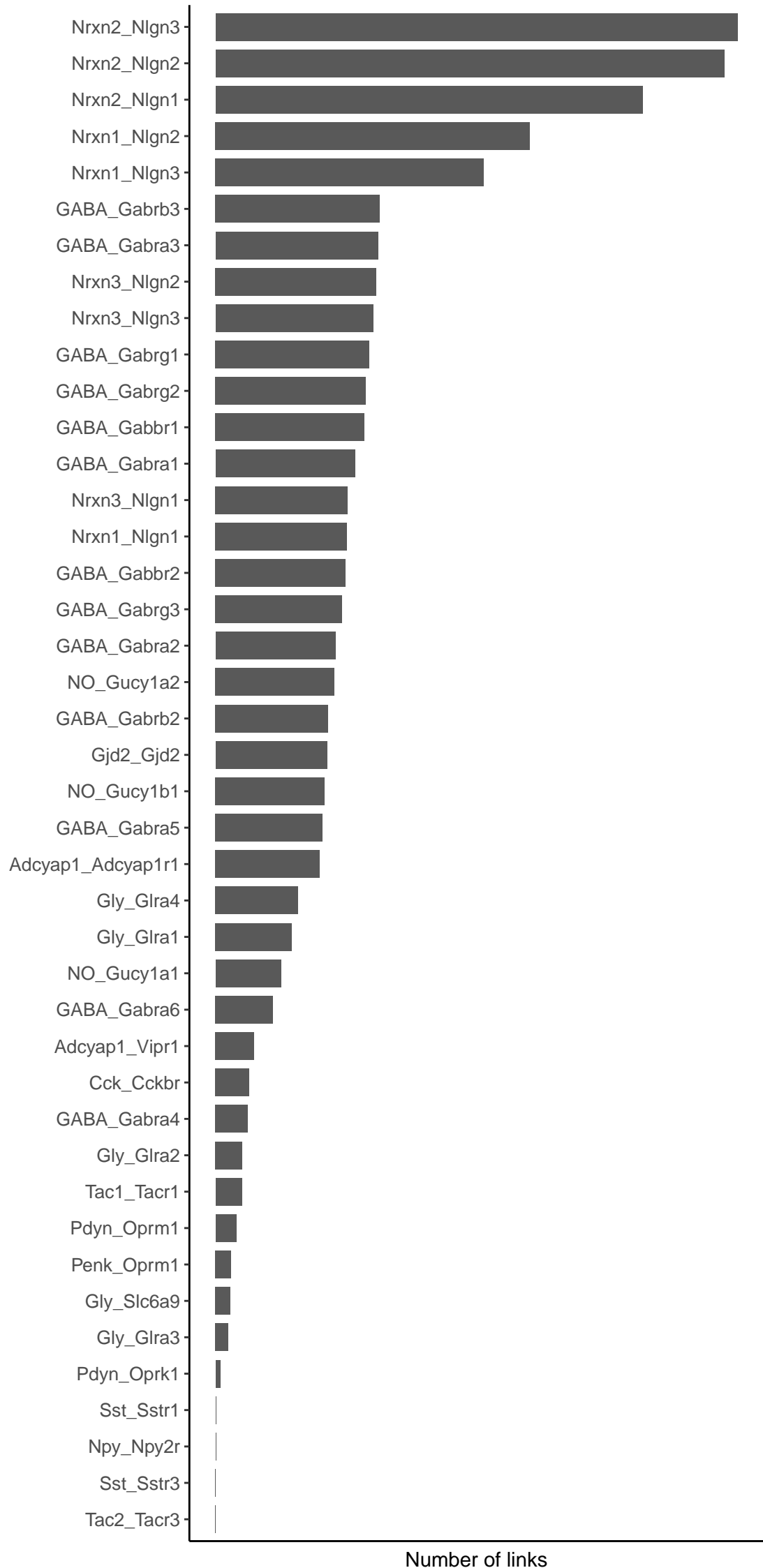

Supplement: Supplementary file 8 — Supplementary Material 8 [file 13293_2024_632_MOESM8_ESM.pdf]

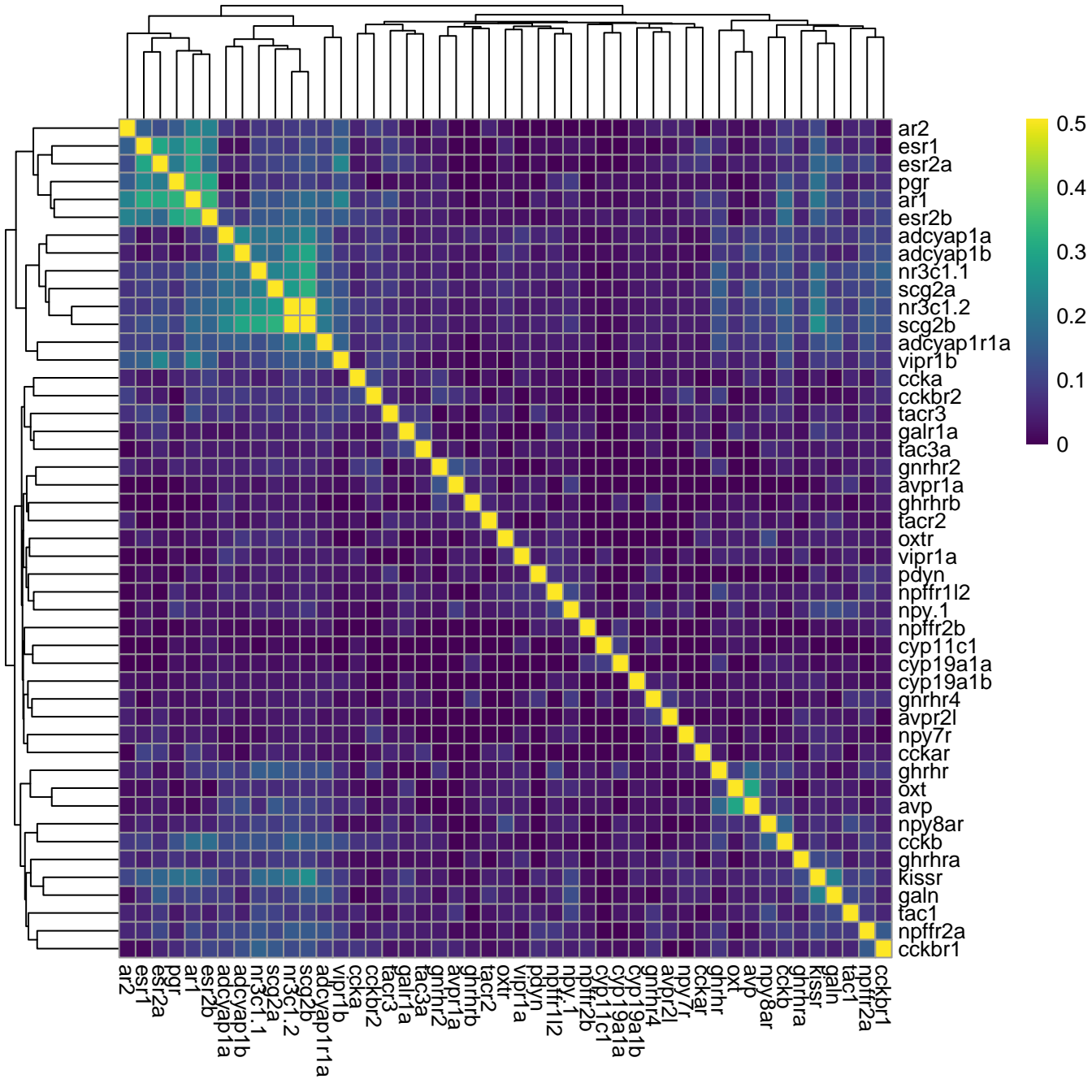

Supplement: Supplementary file 9 — Supplementary Material 9 [file 13293_2024_632_MOESM9_ESM.pdf]

## Sex-DEGs by radial glial subcluster

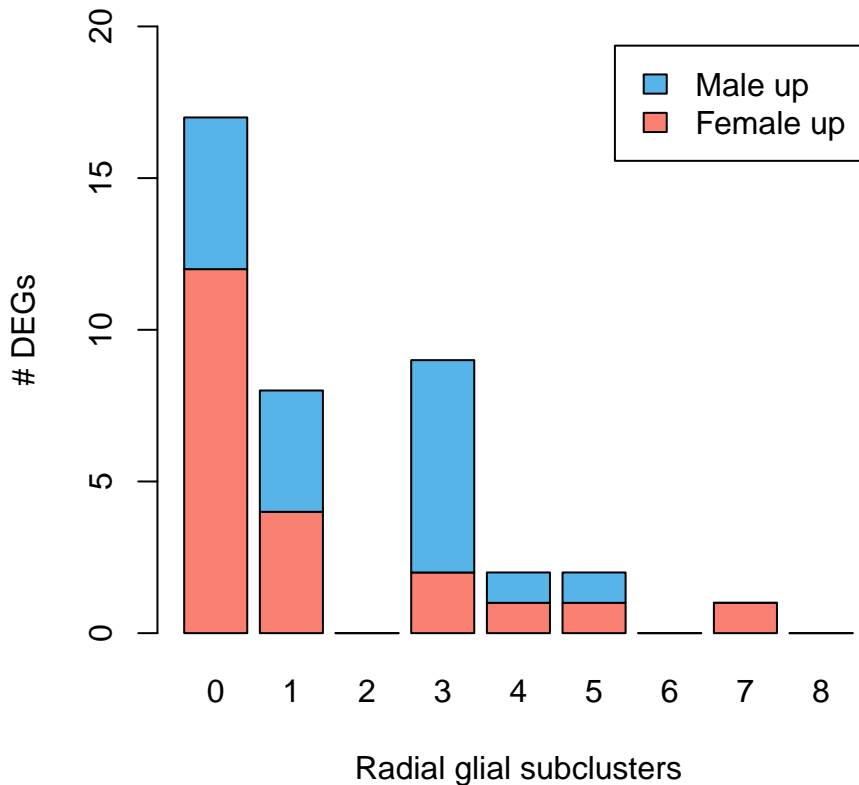

Supplement: Supplementary file 11 — Supplementary Material 11 [file 13293_2024_632_MOESM11_ESM.pdf]

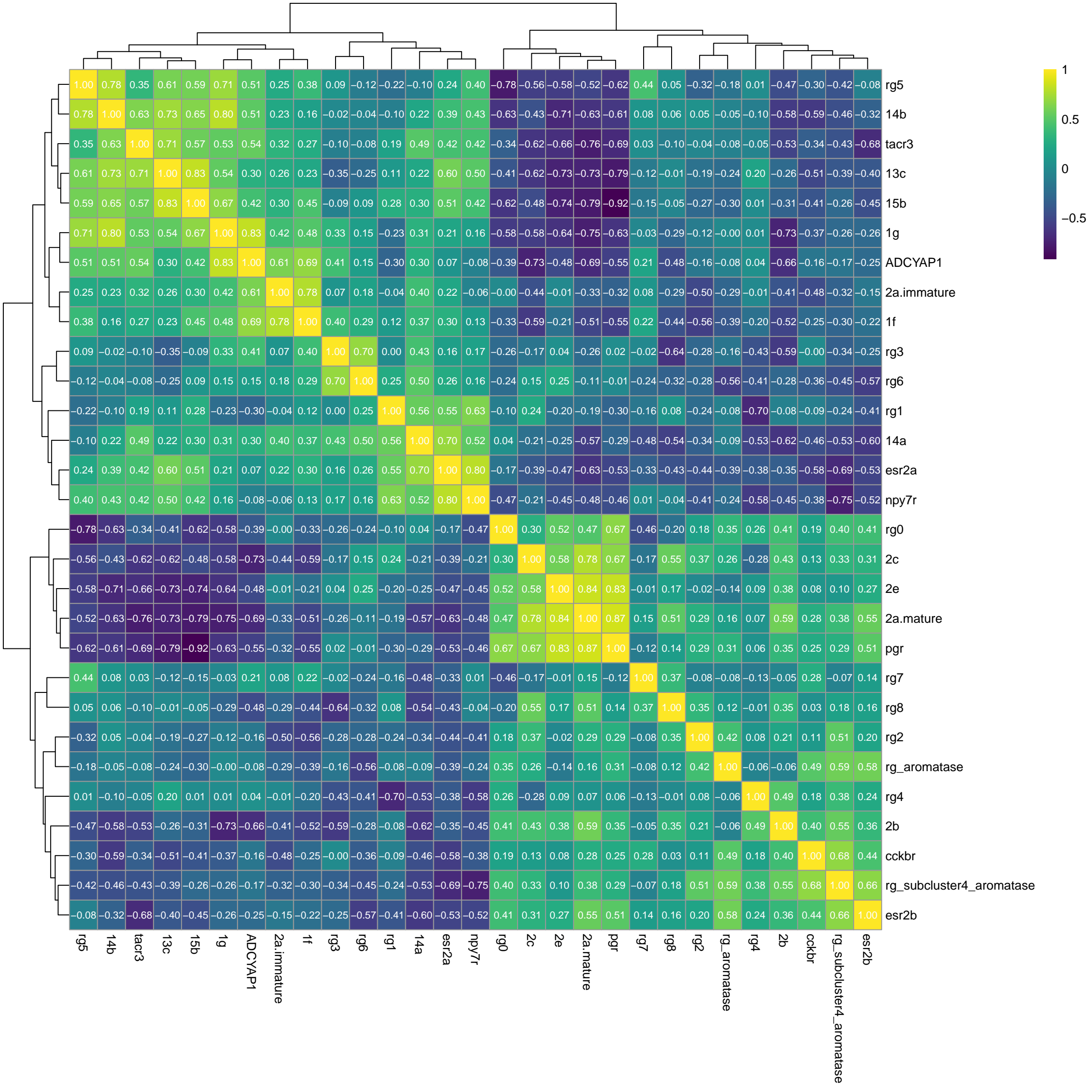

Supplement: Supplementary file 12 — Supplementary Material 12 [file 13293_2024_632_MOESM12_ESM.pdf]
