# Supplementary material for: Adult sex change leads to extensive forebrain reorganization in clownfish
Source: Biol Sex Differ. 2024 Jul 23;15:58. doi: 10.1186/s13293-024-00632-0 (PMC11267845; doi:10.1186/s13293-024-00632-0)
Supplement: Supplementary file 10 — Supplementary Material 10 [file 13293_2024_632_MOESM10_ESM.pdf]

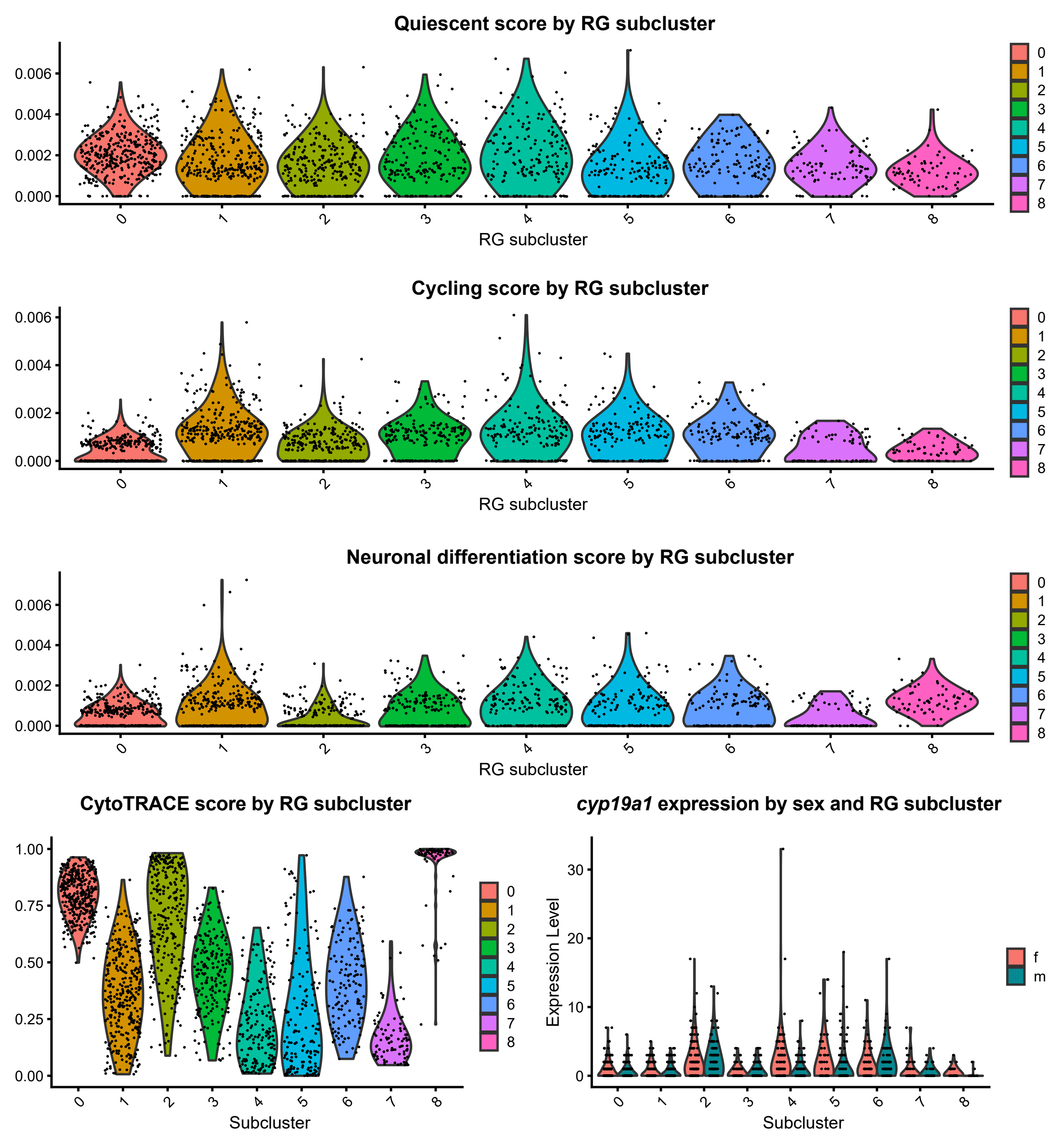
Quiescent score by RG subcluster
Cycling score by RG subcluster
Neuronal differentiation score by RG subcluster
RG subcluster
0.000
0.002
0.004
0.006
0
1
2
3
4
5
6
7
8
CytoTRACE score by RG subcluster
0.00
0.25
0.50
0.75
1.00
Subcluster
cyp19a1 expression by sex and RG subcluster
Expression Level
0
10
20
30
f
m
